# Relationships Between Lateral Ventricle Size, Cerebrospinal Fluid Dynamics, and Aqueductal Resistance in Young Healthy Adults

**DOI:** 10.1002/jmri.70139

**Published:** 2025-10-03

**Authors:** Pan Liu, Jiachen Xie, Kimi Owashi, Yann Attekeble, Jean‐Marc Constans, Cyrille Capel, Olivier Balédent

**Affiliations:** ^1^ Medical Image Processing Department CHU Amiens‐Picardie University Hospital Amiens France; ^2^ CHIMERE Ur 7516 Jules Verne University of Picardy Amiens France; ^3^ Oncology Department CHU Amiens‐Picardie University Hospital Amiens France; ^4^ University of Lille Lille France; ^5^ Radiology Department CHU Amiens‐Picardie University Hospital Amiens France; ^6^ Neurosurgery Department CHU Amiens‐Picardie University Hospital Amiens France

**Keywords:** aqueduct resistance, CSF dynamics, hydrocephalus, phase contrast MRI, ventricular morphology

## Abstract

**Background:**

Ventricular enlargement and abnormal cerebrospinal fluid (CSF) circulation are closely associated in communicating hydrocephalus (NPH), yet their causal relationship remains unclear. Studying healthy populations may help clarify these mechanisms. Existing metrics for CSF dynamics and ventricular morphology are limited by physiological variability such as heart rate and brain size, and aqueductal resistance has been little studied in healthy cohorts.

**Purpose:**

To quantify aqueductal resistance and two ratio‐based indices—the CSF stroke volume ratio of the aqueduct to the cervical region (Ratio‐SV) and the lateral ventricle to total brain area ratio (Ratio‐Area)—in a healthy population, and to examine their interrelationships.

**Study Type:**

Prospective.

**Population:**

34 healthy young adults (17 female, 17 male; age, 25.2 ± 3.9 years old); 4 NPH patients (2 female, 2 male; age, 50–80 years old).

**Field Strength/Sequence:**

3 T MRI with transverse 3D T1‐weighted gradient echo, sagittal 3D Balanced Fast Field Echo (BFFE; gradient echo), and 2D CINE Phase Contrast (CINE‐PC; gradient echo) sequences.

**Assessment:**

Aqueductal resistance was measured on BFFE, Ratio‐Area on T1‐weighted images, and Ratio‐SV on CINE‐PC. Sex differences were also examined.

**Statistical Tests:**

Wilcoxon test was used for group comparisons, and Spearman's correlation for associations among parameters, with *p* < 0.05 considered significant.

**Results:**

In healthy adults, mean aqueductal resistance was 72 ± 42 mPa s/mm^3^, Ratio‐SV 6.0% ± 2.3% and Ratio‐Area 4.5% ± 1.6%. Males exhibited a significantly lower Ratio‐Area compared to females (3.9% ± 1.3% vs. 5.2% ± 1.7%). Ratio‐SV was unaffected by the cardiac cycle. Aqueductal resistance showed a strong negative correlation with Ratio‐SV (*r* = −0.65) but showed no significant correlation with Ratio‐Area (*r* = −0.27). Ratio‐SV and Ratio‐Area were also uncorrelated (*r* = 0.08).

**Data Conclusion:**

Ratio‐based metrics provide useful parameters for evaluating both ventricular morphology and CSF dynamics by reducing the influence of physiological variations. Combined with aqueductal resistance, these baseline data in healthy adults may contribute to a better understanding of the pathophysiology of NPH.

**Evidence Level:**

2.

**Technical Efficacy:**

Stage 1.

## Plain Language Summary

The brain contains clear fluid called cerebrospinal fluid (CSF), which moves through a narrow canal known as the aqueduct. Both CSF flow and the size of the brain's ventricles can be influenced by factors like heart rate or brain size. In this study, we introduced two ratio‐based measures to better capture CSF dynamics and ventricular morphology, and we also combined these with aqueductal resistance. By studying healthy young adults, we identified meaningful relationships among these three parameters. These baseline data provide insights into normal brain physiology and may guide future research on hydrocephalus.

## 1 Introduction

Normal pressure hydrocephalus (NPH) is a form of communicating hydrocephalus, characterized by a clinical triad of gait disturbance, urinary incontinence, and cognitive impairment [1, 2, 3]. The key imaging features of NPH reflect changes in three key parameters: (1) aqueductal dilatation (indicating reduced aqueductal resistance); (2) increased aqueductal cerebrospinal fluid (CSF) stroke volume (SV‐aq) (the volume of CSF oscillating through the aqueduct during a single cardiac cycle) and (3) enlargement of the lateral ventricles [4, 5, 6, 7]. Understanding their temporal sequence and relationships is important for elucidating NPH pathogenesis [8, 9].

However, in patients with NPH, these abnormalities are already present, making it difficult to establish their causal pathways, whether ventricular enlargement causes CSF flow abnormalities or vice versa remains unclear [10, 11]. To address this, a systematic investigation in healthy populations is needed to clarify these ambiguities. Such baseline data may provide useful reference values for understanding CSF–ventricular interactions in healthy adults and could serve as a foundation for future patient‐based studies.

Quantifying and clinically applying these three parameters also pose several challenges. First, although aqueductal resistance is important to understanding CSF circulation, its assessment typically requires intricate, time‐consuming post‐processing of imaging data [12, 13]. Consequently, studies that concurrently evaluate aqueductal resistance, SV‐aq, and area of the lateral ventricles (Area‐vent) remain limited. Second, the absolute values of Area‐vent and SV‐aq are influenced by physiological factors [14]. For example, SV‐aq varies with heart rate [15], while Area‐vent may vary with total brain volume [16, 17]. These physiological influences reduce the reliability of the measures as standalone diagnostic markers [10, 18].

We hypothesized that ratio‐based parameters might reduce inter‐individual variability, improving the comparability of CSF flow and ventricular morphology across populations. Specifically, the CSF stroke volume ratio (Ratio‐SV) is defined as the SV‐aq relative to the CSF at the C2–C3 spinal level (SV‐cv), while the ratio of these two measures (Ratio‐Area) is calculated by dividing the lateral ventricle area by the total brain area.

This study aims to quantify the Ratio‐SV, Ratio‐Area, and aqueductal resistance in healthy young adults with two primary objectives: first, to establish the clinical utility of these novel ratio‐based indices as reliable markers of CSF dynamics and ventricular morphology; second, to explore their interrelationships, providing baseline references that may support future investigations in NPH.

## 2 Materials and Methods

This study was approved by the local Institutional Review Board (CPP Nord Ouest II; reference: PI2019_843_0056) and conducted in accordance with the Declaration of Helsinki. All participants, including healthy volunteers and NPH patients, received a comprehensive explanation of the study's objectives and procedures and provided written informed consent.

### 2.1 Participants

A total of 39 healthy young adults (19 female, 20 males; age, 25.4 ± 3.8 years old; range, 19–35 years old) were enrolled. Exclusion criteria included: (1) contraindications to MRI (e.g., metallic implants or severe claustrophobia); (2) a history of cerebrovascular or respiratory disease, and (3) structural abnormalities detected during a preliminary clinical MRI examination (e.g., tumors, malformations, infarcts, or other significant lesions).

Additionally, data from four NPH patients (2 males, 2 females; age 50–80 years old), recently diagnosed and scanned at our institution, were included for illustrative comparison with the healthy cohort.

The MRI acquisitions lasted approximately 30 min.

### 2.2 MRI Acquisition

Participants underwent imaging in a supine position using a 3T MRI scanner (Philips Achieva; maximum gradient = 80 mT/m; slew rate = 120 mT m^−1^ ms^−1^) equipped with a 32‐channel head coil. During imaging, participants maintained free breathing. Four image series were acquired using three different MRI sequences.

#### 2.2.1 3D T1‐Weighted Sequence

**FIGURE 1:**
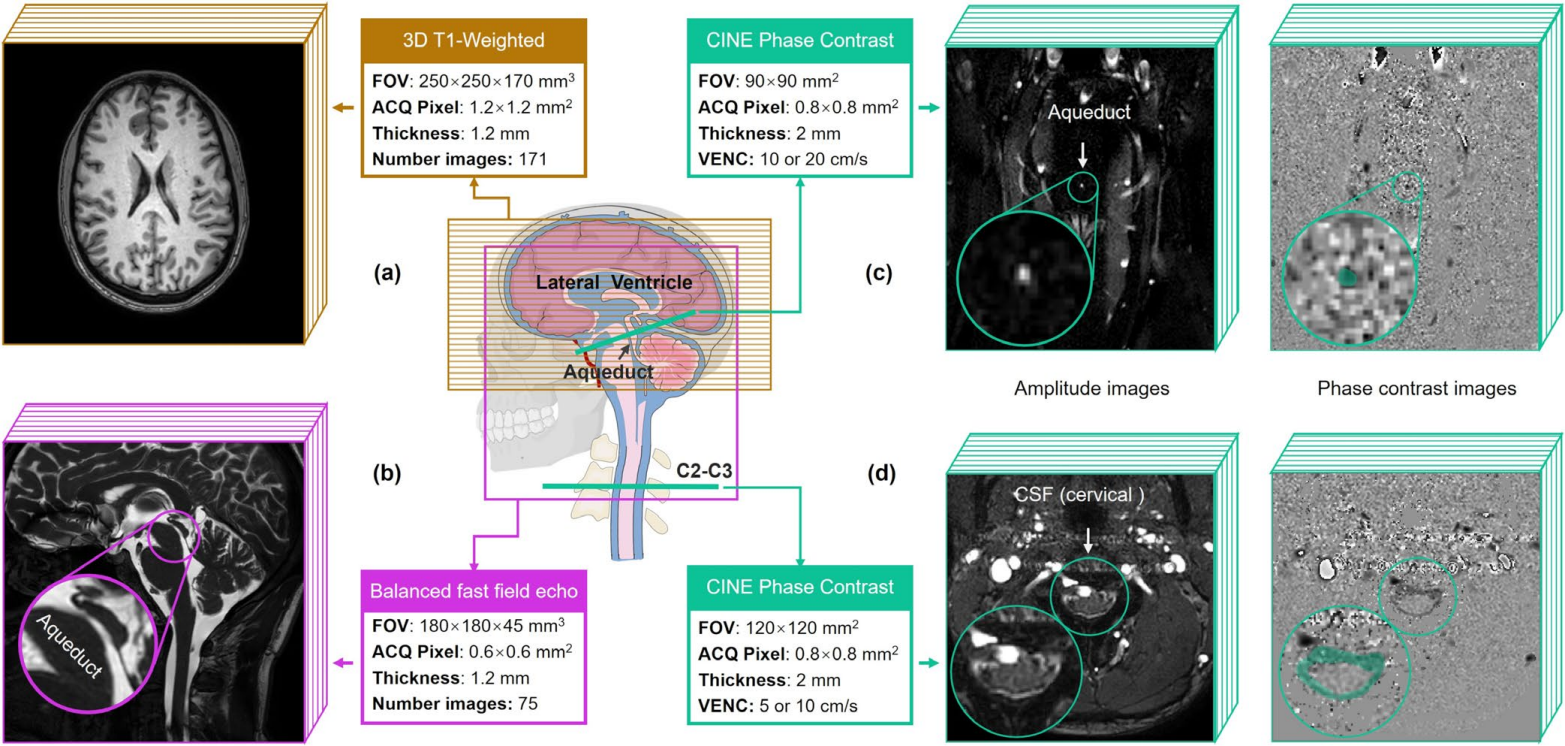
Examples of the three MRI acquisition sequences. A transverse 3D T1‐weighted sequence (a) covering the entire brain was used to calculate ventricular and brain areas. A sagittal Balanced Fast Field Echo sequence (b) was employed to calculate aqueductal resistance. Two CINE Phase Contrast sequences were used to calculate CSF flow at the aqueduct (c) and C2–C3 (d) level. ACQ, acquisition; FOV, field of view; VENC, velocity encoding.

A transverse 3D T1‐weighted gradient echo sequence covering the entire brain (Figure 1a) was employed to quantify Ratio‐Area. T1‐weighted imaging enables fast acquisition while providing relatively high‐resolution anatomical structures with strong contrast. In these images, the ventricles appear dark, and the brain tissue appears bright, facilitating accurate delineation of the lateral ventricle boundaries. The key imaging parameters were as follows: echo time (TE) = 3.5 ms, repetition time (TR) = 7.9 ms, flip angle = 8°, field of view (FOV) = 250 × 250 × 170 mm^3^, in‐plane spatial resolution = 1.2 × 1.2 mm^2^, slice thickness = 1.2 mm, slice gap = 1 mm, acquisition time = 138 s, sensitivity encoding (SENSE) factor = 2, reconstructed spatial resolution = 1 × 1 mm^2^, and number of images = 171.

#### 2.2.2 3D Balanced Fast Field Echo Sequence

A sagittal 3D Balanced Fast Field Echo (BFFE) gradient echo sequence was employed for high‐resolution and high‐contrast morphological imaging of the aqueduct (Figure 1b) to calculate aqueductal resistance. The key imaging parameters were as follows: TE = 2.2 ms, TR = 5.5 ms, flip angle = 45°, FOV = 180 × 180 × 45 mm^3^, in‐plane spatial resolution = 0.6 × 0.6 mm^2^, slice thickness = 1.2 mm, slice gap = 0.6 mm, acquisition time = 183 s, SENSE factor = 1.5, reconstructed spatial resolution = 0.35 × 0.35 mm^2^, and number of images = 75.

#### 2.2.3 CINE Phase Contrast Sequence

2D CINE phase contrast (CINE‐PC) gradient echo sequences were performed to quantify CSF flow at the cerebral aqueduct (Figure 1c) and at the C2–C3 level (Figure 1d). BFFE images were used for localization, ensuring that both slices were positioned perpendicular to the CSF flow direction. At the C2–C3 level, the imaging plane was oriented orthogonal to the spinal subarachnoid space. At the aqueduct level, the plane was placed through the mid‐aqueduct, perpendicular to its longitudinal axis. Fourth‐to‐third ventricle direction and caudal‐cranial direction were designated as positive flow directions. CINE‐PC employed Cartesian k‐space filling, compensated reconstruction, and a finger volumetric plethysmograph as cardiac gating. A total of 32 phase‐contrast images were generated to represent CSF dynamics over an averaged cardiac cycle. The key imaging parameters were as follows: TE = 7–8 ms, TR = 12–15 ms, flip angle = 30°, acquisition time = 56–153 s, and SENSE factor = 1.5. For the aqueduct level, the parameters were: velocity encoding (VENC) = 10 or 20 cm/s, FOV = 90 × 90 mm^2^, in‐plane spatial resolution = 0.5 × 0.5 mm^2^, slice thickness = 2 mm, and reconstructed spatial resolution = 0.38 × 0.38 mm^2^. For the C2–C3 level, the parameters were: VENC = 5 or 10 cm/s, FOV = 120 × 120 mm^2^, in‐plane spatial resolution = 0.8 × 0.8 mm^2^, slice thickness = 3 mm, and reconstructed spatial resolution = 0.5 × 0.5 mm^2^.

### 2.3 Image Post‐Processing

Image post‐processing was performed using a custom tool developed in IDL (Interactive Data Language) [19]. All post‐processing was performed by J.X. (medical student in clinical training, one year of experience in neuroimaging post‐processing). The results were reviewed and confirmed by the first author (P.L., PhD in biomedical engineering, 8 years' MRI analysis experience) and a neurologist co‐author (C.C., MD). The post‐processing workflows and parameter calculation methods for the three types of sequences were as follows:

#### 2.3.1 Calculation of Ratio‐Area

**FIGURE 2:**
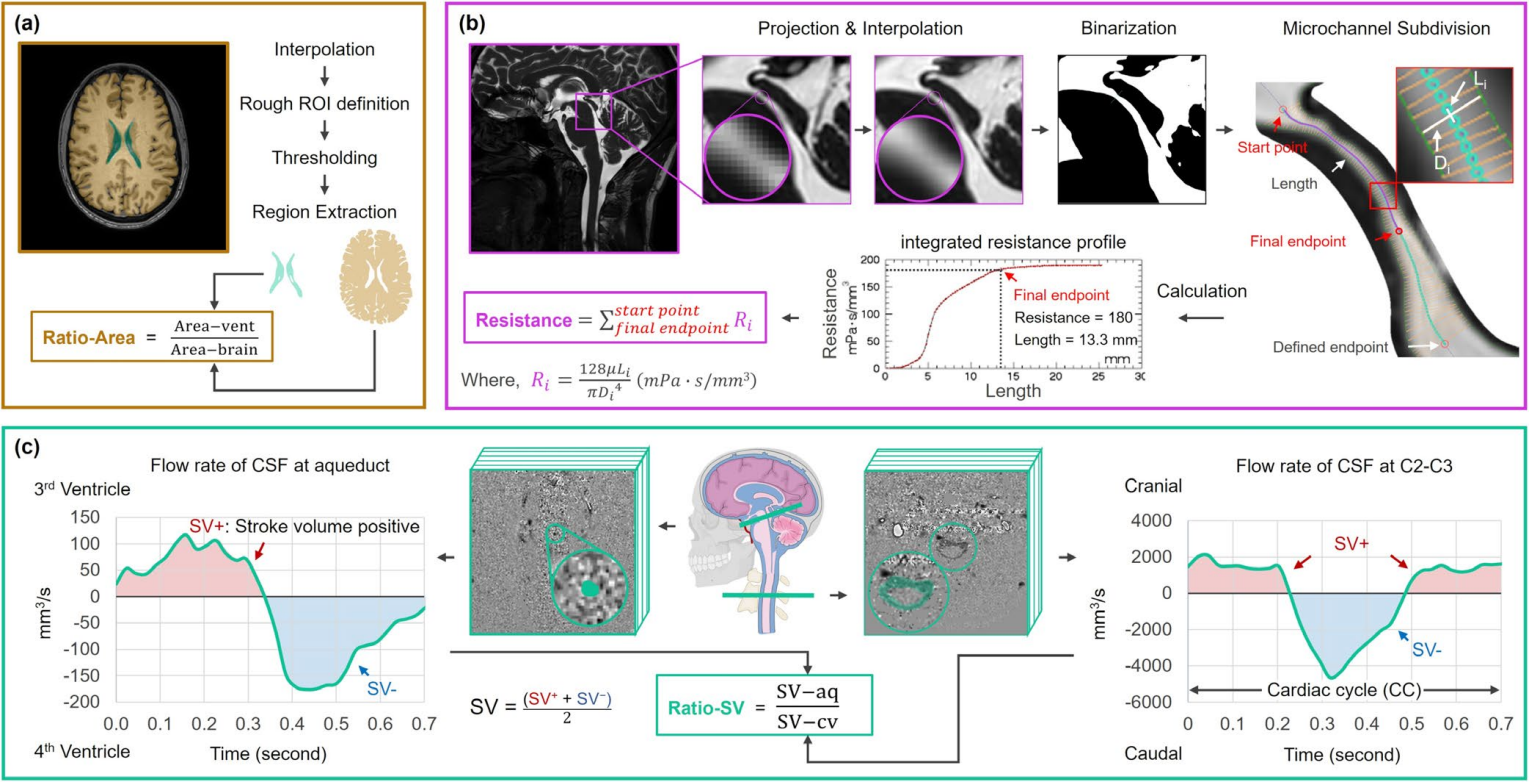
Post‐processing workflow. (a) The T1‐weighted slice with the largest ventricular area ratio was linearly interpolated to enhance resolution. A rough segmentation of the ventricles and brain was performed, followed by threshold‐based refinement to obtain precise ventricular (Area‐vent) and brain (Area‐brain) areas. The Ratio‐Area was calculated as their ratio. (b) Balanced Fast Field Echo (BFFE) slices containing the aqueduct underwent Maximum Intensity Projection (MIP), followed by linear interpolation and semi‐automatic binarization. The aqueduct's start and endpoints were manually defined, and the software performed finite element subdivision, computing segmental resistance using Poiseuille's equation to derive the total aqueductal resistance. The 95% integrated resistance point was selected as the final endpoint. Morphological parameters and resistance values were recorded. (c) Phase‐contrast MRI slices at the aqueduct and C2–C3 level underwent semi‐automatic segmentation and automated background phase correction to generate flow rate curves. Stroke volumes (SV‐aq and SV‐cv) were obtained as the mean of SV^+^ and SV^−^, where SV^+^ and SV^−^ were computed by integrating the flow rate over time in the corresponding directions. Ratio‐SV was then determined as the ratio of SV‐aq to SV‐cv.

The Ratio‐Area was calculated in four steps:

1. Linear Interpolation: T1‐weighted images underwent linear interpolation, increasing the reconstructed spatial resolution to 0.1 × 0.1 mm^2^ to enhance segmentation accuracy.
2. Semi‐Automatic Segmentation: A semi‐automatic segmentation method was used to extract the ventricular area (Area‐vent) and brain area (Area‐brain). The operator manually selected the slice with the largest ventricular proportion and outlined rough ROIs for the ventricles and the whole brain (including both gray and white matter). The threshold was then defined using one of the following approaches: detecting the maximum intensity gradient along a user‐drawn line, selecting from automatic binarization algorithms, or manually adjusting the threshold—a commonly used method that offered a good balance between accuracy and visual consistency—to achieve a smooth and complete boundary (Figure S1). Area‐vent was defined as the segmented ventricular area corresponding to pixels labeled 0 within the ventricular ROI, with the choroid plexus (labeled 1) excluded. Area‐brain was defined as the segmented brain area corresponding to pixels labeled 1 within the brain ROI, also excluding the choroid plexus.
3. Ratio‐Area Determination: The Ratio‐Area was calculated as the ratio of Area‐vent to Area‐brain. The slice with the maximal Ratio‐Area was visually identified by J.X. and independently verified by P.L. If their selections differed, quantitative measurements were used to confirm the slice with the highest Ratio‐Area (Figure 2a).
4. Data Recording: The Ratio‐Area, Area‐vent, and Area‐brain of the selected slice were recorded.

#### 2.3.2 Calculation of Aqueductal Resistance

The aqueductal resistance was calculated in five steps [20]:

1. Interpolation & projection: The BFFE images underwent linear interpolation, increasing the spatial resolution to 0.03 × 0.03 mm^2^. Maximum intensity projection (MIP) was then applied to obtain the aqueduct projection image.
2. Binarization: A line was manually drawn at the narrowest point of the aqueduct to guide thresholding, as aqueductal resistance is most sensitive to the minimal radius. Our custom post‐processing software automatically detected the maximum intensity gradient along the pixel intensity profile of this line and used the corresponding pixel intensity as the binarization threshold.
3. Aqueduct Segmentation: The co‐author (J.X., medical extern) manually defined the start point at the distal end of the third ventricle and the end point at the anterior portion of the fourth ventricle (user‐defined aqueduct length). The software then generated the aqueductal contour and its central trajectory, dividing the aqueduct into 100 microchannel segments (Li) and calculating the resistance of each microchannel (Ri) based on Poiseuille's formula:

$$\mathrm{Ri}=\frac{128\cdot \mu \cdot \mathrm{Li}}{\pi \cdot {\mathrm{Di}}^{4}}\left(\mathrm{mPa}\;s/{\mathrm{mm}}^{3}\right)$$

where *μ* represents the dynamic viscosity, which was set to 0.71 mPa s in this study (corresponding to the viscosity of water at 36°C). Li denotes the length of the microchannel, and Di represents the local diameter at the central point of each microchannel segment.

4 Endpoint Standardization: To minimize operator‐dependent variability in defining the distal endpoint of the aqueduct (anterior fourth ventricle), the software automatically set the final endpoint at the 95% cumulative point of the integrated resistance profile (cumulative sum of Ri along the aqueduct). This method reduces the influence of the wide, low‐resistance distal segment, yielding a standardized “effective length” that better reflects the functional distribution of resistance (Figure 2b).
5 Parameter Recording: The software calculated and recorded the aqueductal resistance, user‐defined length, length, mean diameter, and minimum diameter.

#### 2.3.3 Calculation of Ratio‐SV

The Ratio‐SV was calculated in three steps:

1. Semi‐Automatic Segmentation: A semi‐automatic segmentation method based on velocity spectrum characteristics was applied to define fixed regions of interest (ROIs) for CSF at the aqueduct and C2–C3 levels in the two CINE‐PC series [21]. Flow rate curves, containing 32 sampling points, were extracted as the product of ROI area and the mean velocity within the ROI.
2. Background Field Correction: A fully automated background field correction was performed to compensate for velocity offsets [22]. This was achieved by identifying static tissue surrounding the target CSF region based on magnitude image intensity and velocity characteristics, setting its mean velocity as the new zero‐velocity reference. Additionally, the software incorporated a de‐aliasing correction for instances where CSF velocity exceeded the predefined velocity encoding (VENC).
3. Calculation of SV and Ratio‐SV: The software computed SV‐aq (stroke volume at the aqueduct) and SV‐cv (stroke volume at the C2–C3 level) from the corrected flow rate curves. SV was calculated as the mean of SV positive and SV negative, obtained by integrating the flow rate over time in the positive and negative directions, respectively. This represents the volume of CSF flowing into or out of the plane during a single cardiac cycle. Ratio‐SV was then determined as the ratio of SV‐aq to SV‐cv (Figure 2c).

### 2.4 Statistical Analysis

Statistical analyses were performed using SPSS (version 26), and statistical plotting was conducted in Origin (version 2022). Data were presented as mean ± standard deviation (SD), interquartile range (Q1–Q3, 25th–75th percentile), and coefficient of variation (CV%). To improve the robustness of baseline metrics and reduce their influence on correlation analyses, outliers were excluded when any of the three core parameters—Ratio‐Area, Ratio‐SV, or aqueduct resistance—exceeded three times the interquartile range from the first or third quartile (IQR). Group differences between male and female participants were assessed using Wilcoxon rank‐sum tests for unpaired data. Correlations between variables were evaluated using Spearman's rank correlation test. All tests were two‐tailed, with statistical significance set at *p* < 0.05.

## 3 Results

Five participants were excluded from the analysis: two due to motion artifacts in BFFE imaging, one due to *a* > 10% cardiac cycle mismatch between the two CINE‐PC slices, and two identified as outliers based on the 3 × IQR criterion—one with an abnormally high aqueductal resistance (301 mPa s/mm^3^) and one with an elevated Ratio‐SV (18.8%). Ultimately, 34 participants were included in data analysis. The cardiac cycle difference between the two CINE‐PC slices was 1.1% (range: −5.7% to 8.6%). The age distribution between 17 males and 17 females showed no statistically significant difference (26.2 ± 4.3 vs. 24.2 ± 3.3 years old, *p* = 0.24).

### 3.1 Parameter Distribution and Sex Differences

Key parameters, including Ratio‐Area, aqueductal resistance, and Ratio‐SV, were analyzed (Table 1). The mean values were 4.5% ± 1.6% for Ratio‐Area, 72 ± 42 mPa s/mm^3^ for aqueductal resistance, and 6.0% ± 2.3% for Ratio‐SV. Aqueductal resistance showed the highest variability (CV = 59%), followed by Ratio‐SV (CV = 38%) and Ratio‐Area (CV = 35%). Area‐vent showed greater variability than Area‐brain (CV: 32% vs. 8%), and SV‐aq demonstrated higher variability than SV‐cv (CV: 46% vs. 24%).

**TABLE 1:** Parameter distribution and sex‐specific differences.

	Area‐vent	Area‐brain	Ratio‐Area	Length	D‐min	D‐mean	Resistance	Cardiac cycle	SV‐aq	SV‐cv	Ratio‐SV
	(mm^2^)	(mm^2^)	(%)	(mm)	(mm)	(mm)	(mPa s/mm^3^)	(ms)	(mm^3^)	(mm^3^)	(%)
Total (*n* = 34)											
Mean ± SD	6.5 ± 2.1	146 ± 11	4.5 ± 1.6	15.4 ± 2.9	1.2 ± 0.2	1.9 ± 0.3	72 ± 42	825 ± 136	35 ± 16	598 ± 143	6.0 ± 2.3
Q1–Q3	4.9–8.5	137–154	3.3–6.2	13.0–18.2	1.0–1.4	1.7–2.0	43–77	741–876	21–46	477–681	4.4–7.3
CV	32%	8%	35%	19%	19%	14%	59%	17%	46%	24%	38%
Male (*n* = 17)											
Mean ± SD	5.8 ± 1.7	151 ± 10	3.9 ± 1.3	16.6 ± 2.8	1.3 ± 0.2	2.0 ± 0.2	62 ± 38	862 ± 151	41 ± 18	595 ± 161	6.9 ± 2.2
Q1–Q3	4.8–7.2	144–158	3.1–4.9	14.8–18.6	1.1–1.5	1.9–2.1	37–70	767–891	29–46	476–725	6.1–8.7
CV	29%	7%	33%	17%	19%	9%	61%	18%	44%	27%	32%
Female (*n* = 17)											
Mean ± SD	7.2 ± 2.2	140 ± 9	5.2 ± 1.7	14.2 ± 2.5	1.2 ± 0.2	1.8 ± 0.3	82 ± 45	788 ± 112	30 ± 13	600 ± 127	5.1 ± 2.0
Q1–Q3	5.4–9.1	137–147	3.7–6.4	12.5–15.7	1.0–1.3	1.6–1.8	55–85	741–851	19–36	516–673	2.7–6.8
CV	31%	7%	32%	18%	18%	18%	55%	14%	43%	21%	39%
Male vs. female											
*Z* value	−1.93	2.65	−2.17	2.45	1.83	2.82	−1.89	1.24	1.76	−0.07	2.27
Effect size (*r*)	−0.33	0.45	−0.37	0.42	0.31	0.48	−0.32	0.21	0.30	−0.01	0.39
*p* value	0.054	0.008**	0.030*	0.014*	0.068	0.005**	0.058	0.215	0.079	0.945	0.023*

*Note*: Statistical differences between male and female were determined using the Wilcoxon rank‐sum test, with *p < 0.05 and **p < 0.01, *Z* value indicating the deviation of the observed rank‐sum from its expected value under the null hypothesis, the effect size *r* is calculated as the *Z* value divided by the square root of the total sample size (*n* = 36).

Abbreviations: Area‐brain, brain area; Area‐vent, ventricular area; CC, mean cardiac cycle duration derived from CSF flow curves; CV, coefficient of variation; D‐Mean, mean aqueductal diameter; D‐Min, minimum aqueductal diameter; L, aqueductal path length from starting to final endpoint; Ratio‐Area, ratio of Area‐vent to Area‐brain; Ratio‐SV, ratio of SV‐aq to SV‐cv; Resistance, aqueductal resistance; SD, standard deviation; SV‐aq, CSF stroke volume at the aqueduct; SV‐cv, CSF stroke volume at the cervical level (C2–C3).

**FIGURE 3:**
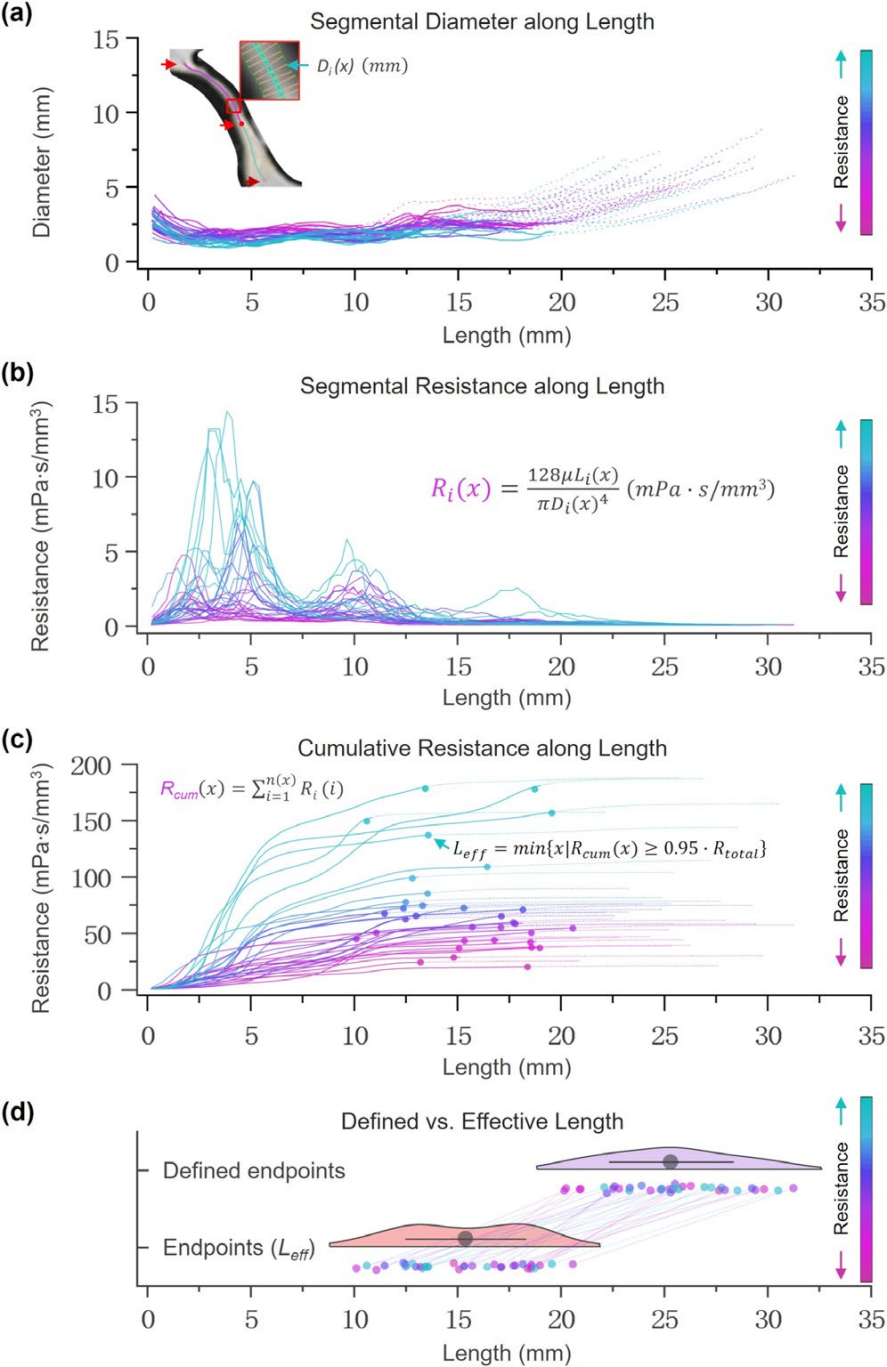
Morphological and resistance‐based characterization of the cerebral aqueduct in 34 participants. Color represents total aqueduct resistance, with higher values shown in cyan and lower values in magenta. (a) Segmental diameter profiles along the aqueduct. Solid lines represent the effective length (from start to stop point), while dashed lines indicate excluded segments outside this range (i.e., between user‐defined endpoints and effective limits). (b) Segmental resistance (Ri) computed along the aqueduct length. (c) Cumulative resistance curves derived from the integral of the segmental resistance (panel b). Solid lines correspond to the effective length; dashed lines represent the remaining segments. Dots indicate the point at which 95% of the total resistance is reached, which was used to define the effective length. (d) Comparison between user‐defined aqueduct lengths (top) and effective lengths (bottom).

Figure 3 shows the distribution of aqueduct diameter, segmental resistance, and cumulative resistance along the length. Diameter varied little, while segmental resistance changed more noticeably across positions. The point with the smallest diameter contributed the most to total resistance. The distributions of user‐defined length and effective length were different: user‐defined length showed a near‐normal distribution, while effective length showed a bimodal pattern (Figure 3d).

**FIGURE 4:**
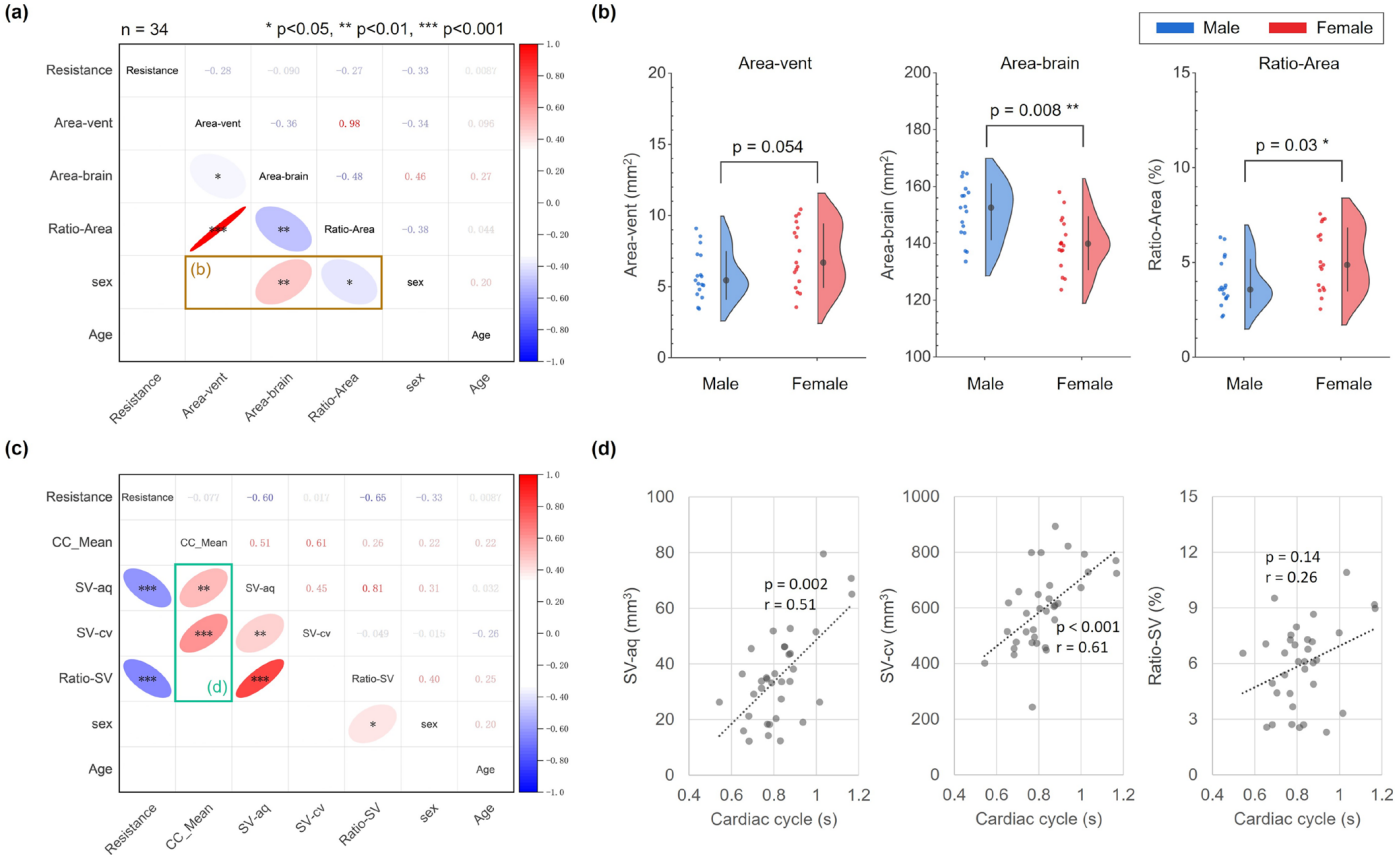
Correlation analyses among morphological and hydrodynamic parameters. (a, c) Spearman correlation matrices: The upper triangle shows Spearman's rank correlation coefficient (*r*), and the lower triangle displays trend ellipses with asterisks indicating three levels of significance (*p* < 0.05, *p* < 0.01, *p* < 0.001). Resistance denotes aqueductal resistance; Area‐vent, lateral ventricular area; Area‐brain, total brain area; and Ratio‐Area, the ratio of Area‐vent to Area‐brain. CC_Mean represents the mean cardiac cycle of CSF flow curves across two planes. SV‐aq and SV‐cv indicate CSF stroke volume at the aqueduct and cervical levels, respectively, while Ratio‐SV represents the ratio of SV‐aq to SV‐cv. (b) Group comparisons of Area‐vent, Area‐brain, and Ratio‐Area between males and females. (d) Scatter plots of SV‐aq, SV‐cv, and Ratio‐SV against cardiac cycle, with corresponding Spearman correlation *p* and *r* values.

Sex‐specific analyses revealed that Ratio‐Area was significantly higher in males than in females, while Area‐vent did not differ significantly between sexes (*p* = 0.054) (Table 1, Figure 4b). Similarly, Ratio‐SV showed significant sex differences, whereas SV‐aq did not (*p* = 0.079) (Figure 4c). Males had significantly greater aqueduct length and average diameter than females, but aqueductal resistance did not differ significantly (*p* = 0.058).

### 3.2 Correlation Analyses of Morphological and Hydrodynamic Parameters

Spearman correlation analysis of morphological parameters showed a significant correlation between Area‐vent and Area‐brain (*r* = −0.36). Age (19–35 years) was not significantly correlated with any morphological parameter (aqueductal resistance: *p* = 0.96; Area‐vent: *p* = 0.59; Area‐brain: *p* = 0.12; Ratio‐Area: *p* = 0.81). Aqueductal resistance was not significantly correlated with either Area‐vent (*p* = 0.11, *r* = −2.8) or Ratio‐Area (*p* = 0.61, *r* = −0.09) (Figure 4a).

Among hydrodynamic parameters, both SV‐aq and SV‐cv were significantly influenced by the cardiac cycle, whereas Ratio‐SV was not (*p* = 0.14) (Figure 4c,d). Age showed no significant correlation with any hydrodynamic parameter (SV‐aq: *p* = 0.86; SV‐cv: *p* = 0.14; Ratio‐SV: *p* = 0.16). Aqueductal resistance exhibited similar correlation strengths with Ratio‐SV and SV‐aq (*r* = −0.65 and *r* = −0.60, respectively).

### 3.3 Comparison With NPH

**FIGURE 5:**
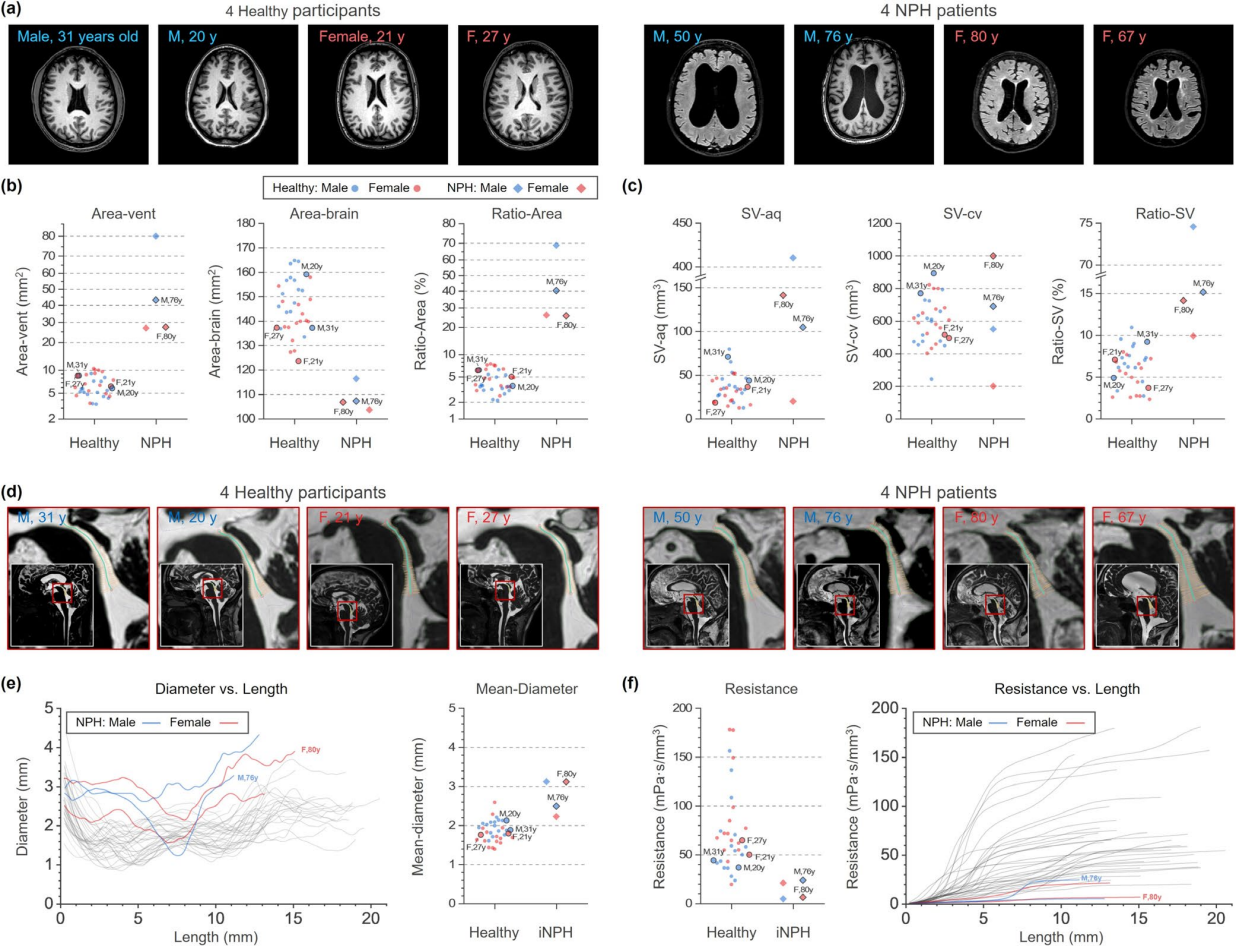
Comparison with NPH patients. (a) Representative axial T1‐weighted images of four healthy participants (left) and four NPH patients (right), with sex and age indicated. These eight individuals were consistently used across panels (b–f) and are highlighted throughout. (b) Group comparisons of lateral ventricular area (Area‐vent), brain area (Area‐brain), and their ratio (Ratio‐Area). Each dot represents one participant; symbol and color indicate group and sex. (c) Stroke volume at the aqueduct (SV‐aq) and cervical spinal canal (SV‐cv), and their ratio (Ratio‐SV), shown for healthy and NPH groups. (d) Sagittal T2‐weighted projection images centered on the aqueduct. Red insets show magnified views of the regions within the white boxes, highlighting the segmented aqueduct between the start point and user‐defined endpoint. (e) Aqueductal diameter profiles along the effective length (start point to endpoint) (left), and mean diameter (right). Colored curves represent individual NPH patients; gray lines represent the healthy group. (f) Effective aqueductal resistance (left) and cumulative resistance curves along the effective length (right).

The Figure 5 shows a visual comparison between four NPH patients and the healthy group. Because the NPH cohort was small (*n* = 4) and significantly older than the healthy group, this comparison was presented only as a visual illustration without statistical analysis. NPH patients had larger Ratio‐Area, lower aqueductal resistance, and reduced Ratio‐SV.

Differences in aqueduct shape were clearly reflected in the diameter profiles (Figure 5e). In healthy participants, the profiles generally exhibited a localized narrowing near the third ventricle, which contributed substantially to the calculated resistance (Figure 3b). In contrast, in NPH patients, the narrowing occurred closer to the mid‐portion of the aqueduct, with both the mean and minimum diameters being larger.

### 3.4 Relationships Between Ratio‐Area, Ratio‐SV and Resistance

**FIGURE 6:**
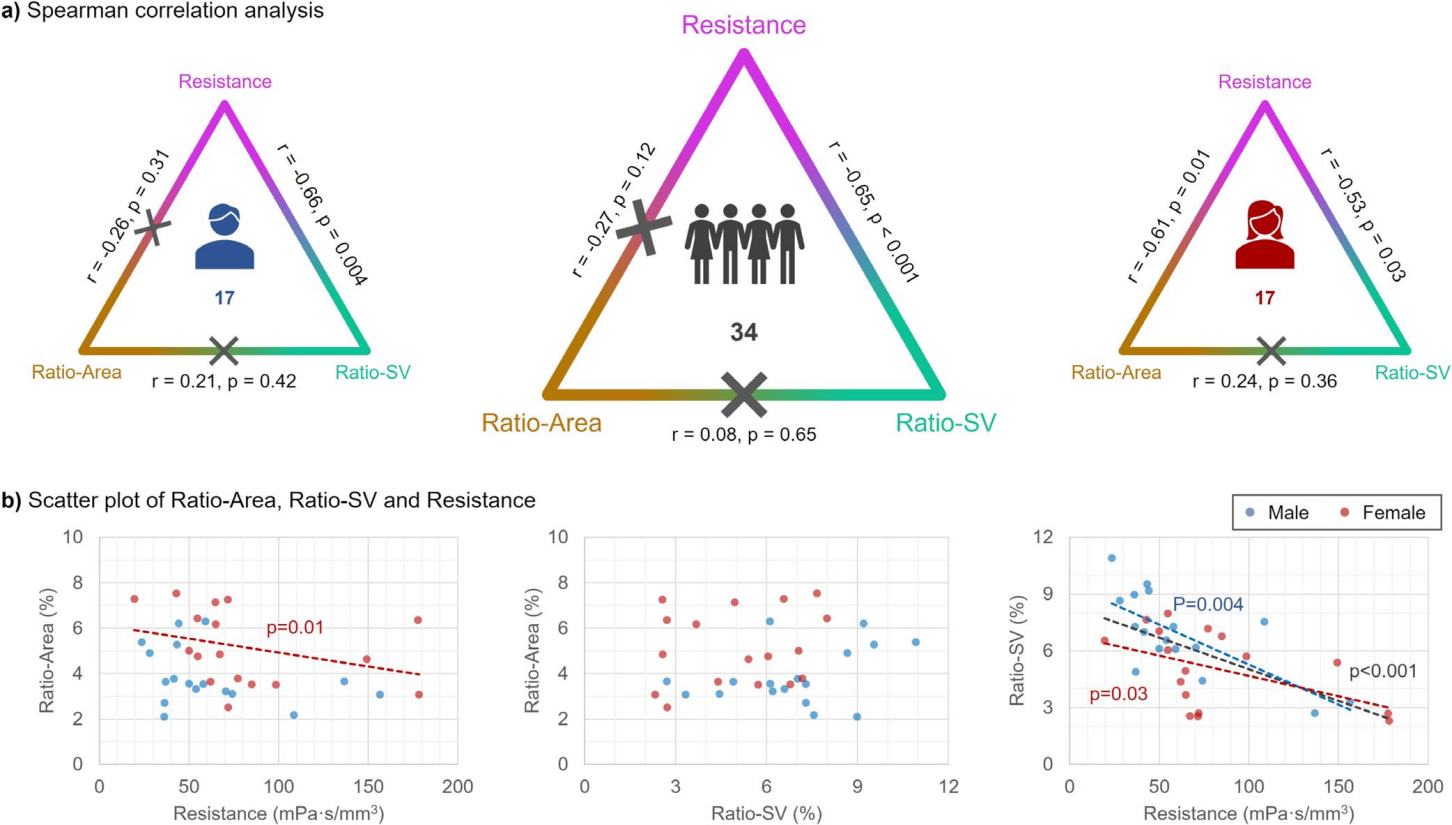
Relationships between Ratio‐Area, Ratio‐SV and Resistance. (a) Spearman correlation results among the three parameters, with gender differentiation. (b) Scatter plots of pairwise relationships between the three parameters, color‐coded by gender. The gray trend line represents the overall trend, and significant correlations are marked with *p* values. Ratio‐Area denotes the proportion of ventricular area to total brain area, and Ratio‐SV denotes the proportion of stroke volume in the aqueduct to that at the cervical level.

As shown in Figure 6, aqueductal resistance significantly correlated negatively with Ratio‐SV in all participants, males alone, and females alone. In contrast, Ratio‐SV and Ratio‐Area showed no significant correlation in all participants (*p* = 0.65), males (*p* = 0.42), or females (*p* = 0.36).

Sex‐specific differences were observed in the correlation between aqueductal resistance and Ratio‐Area. In males, no significant correlation was found (*p* = 0.31), whereas in females, a significant negative correlation was observed.

Additional correlation analyses between aqueductal resistance and the original area‐ and volume‐based parameters (Area‐vent, Area‐brain, SV‐aq, and SV‐cv) are shown in Figure S2.

## 4 Discussion

This study quantified the interrelationships between ventricular morphology and CSF dynamics across cranial and spinal compartments in healthy young adults.

While both ventricular morphology and CSF dynamics are known to be altered in patients with hydrocephalus, our aim was to analyze how these parameters interact in the healthy population in order to infer potential physiological mechanisms that may contribute to the development of ventricular dilatation.

We chose to focus on the axial slice showing the largest ventricular area because it is simple to measure and has been shown to correlate well with total ventricular volume [7]. Given the observed variability in head size across participants, we further hypothesized that accounting for individual brain size could provide more meaningful comparisons; therefore, we introduced a normalized parameter, the Ratio‐Area, to adjust for anatomical differences.

The cerebral aqueduct serves as a strategic anatomical pathway between the ventricular system and the rest of the intracranial CSF compartments. The stroke volume through the aqueduct (SV‐aq) is considered a key clinical parameter and is frequently elevated in NPH [23]. However, SV‐aq alone does not reflect the overall CSF dynamics. To address this, we included the spinal CSF stroke volume (SV‐cv) as an additional parameter and further calculated the ratio SV‐aq/SV‐cv to minimize the influence of cardiac cycle variability and enhance the interpretability of aqueductal flow as a normalized measure.

Finally, since aqueductal CSF flow depends on both pressure gradient and resistance, we also quantified aqueductal resistance in this study. This parameter may serve as an important intermediary between ventricular morphology and CSF dynamics, thereby contributing to a more comprehensive understanding of their interaction in both health and disease.

### 4.1 Ratio‐Area and Ratio‐SV as Useful Physiological Biomarkers

Absolute values, such as Area‐vent and SV‐aq, pose challenges for NPH diagnosis due to inter‐individual variations and physiological fluctuations. This study demonstrated these limitations by showing that Area‐vent was influenced by brain volume differences and that SV‐aq varied with cardiac cycle duration, thereby highlighting the advantages of ratio‐based parameters.

First, Area‐vent may fail to accurately reflect pathological enlargement due to inter‐individual brain volume differences, particularly those caused by sex and age‐related variations [24]. For example, males in this study had significantly larger brain volumes than females, consistent with previous findings [24, 25]. Ratio‐Area exhibited more pronounced sex differences compared to Area‐vent (Table 1), suggesting that it better accounts for inter‐individual variations. Establishing sex‐ and age‐specific reference curves for Ratio‐Area could further enhance its clinical utility.

Second, SV‐aq and SV‐cv are influenced by the cardiac cycle duration, consistent with previous findings [26]. However, factors such as physical activity, stress, fatigue, and postprandial states can dynamically alter heart rate or cardiac function, reducing the reliability of SV‐aq as a diagnostic parameter [18, 27].

In contrast, Ratio‐SV was unaffected by the cardiac cycle and aqueductal resistance correlated more strongly with ratio‐based parameters (Figure 4). Previous studies have also shown that SV‐cv and intracranial blood volume fluctuations do not significantly differ between healthy individuals and NPH patients, whereas the main difference was observed in the SV‐aq [4, 28]. This suggests that Ratio‐SV effectively eliminates the influence of heart rate variability and inter‐individual differences in CSF dynamics, improving its stability as a diagnostic metric.

The results of this study are consistent with previous findings [21], suggesting that SV‐aq contributes minimally to compensating for cardiac‐induced intracranial blood volume fluctuations (Table 1). The major compensatory role is played by the intracranial subarachnoid spaces, particularly the pontine and cerebellar cisterns. This is primarily due to the high resistance of the aqueduct, which limits the amount of CSF that can be displaced through the aqueduct.

These findings underscore the stability of ratio‐based parameters in representing ventricular morphology and CSF dynamics. Future studies should evaluate their clinical sensitivity and specificity for NPH diagnosis and postoperative assessment.

### 4.2 Aqueductal Resistance as an Important Parameter

Aqueductal resistance is an important parameter reflecting the amplitude of CSF exchange between the ventricular system and the subarachnoid spaces. While aqueductal obstruction can lead to ventricular dilation, paradoxically, some patients with enlarged ventricles also exhibit aqueductal enlargement. Therefore, assessing aqueductal resistance is important for both the diagnosis of NPH and the investigation of its underlying pathophysiological mechanisms. However, its application faces quantification challenges. Aqueductal resistance, whether calculated using the Poiseuille equation or Navier–Stokes equations, is highly sensitive to aqueductal diameter variations, necessitating high‐precision finite element subdivision for accurate quantification [20]. As shown in Figure 3a, aqueductal diameter varies along its length rather than remaining constant. Given that resistance is inversely proportional to the fourth power of diameter, even small variations can lead to large differences in segmental resistance (Figure 3b). Moreover, a previous study has shown that aqueductal resistance does not significantly correlate with cross‐sectional area derived from only one slice of low‐resolution phase‐contrast imaging [20]. This highlights the necessity of high‐resolution imaging techniques, such as BFFE in the entire aqueduct length, for reliable quantification.

This study employed an automated platform for finite element subdivision of the aqueduct, capturing its complex geometry and providing accurate resistance distribution while simplifying post‐processing. By defining the endpoint at the 95% cumulative resistance point, this approach reduces variability in length definition and ensures that the effective length better reflects the functional resistance distribution. Taken together, these features facilitate the integration of aqueductal resistance into clinical applications.

Beyond differences in resistance, patients with NPH also showed distinct diameter profiles along the aqueduct (Figure 5e), lacking the localized narrowing commonly seen in healthy individuals. These differences cannot be excluded as potentially related to age, which warrants further investigation, because age‐dependent changes in aqueductal morphology could alter resistance and, in turn, CSF circulation. This underscores the importance of considering the full aqueduct profile, as single‐point measures (e.g., minimum diameter or total length) may not capture relevant morphological variations. Previous studies have also reported cerebral hemodynamic changes in NPH, including reduced cerebral blood flow and impaired vascular compliance [4, 29, 30]. Such alterations are often linked to parenchymal injury and white matter loss, and there is currently insufficient evidence to support cerebral hemodynamic changes as a primary cause of NPH.

Future research could explore the potential application of aqueductal resistance in NPH diagnosis and preoperative planning. Moreover, integrating aqueductal morphology, ventricular morphology, CSF dynamics, and cerebral hemodynamics may provide a more comprehensive assessment of neurofluid interactions.

### 4.3 Baseline Characterization of CSF Circulation and Ventricular Morphology in Healthy Adults

The pathogenesis of communicating hydrocephalus remains unclear. CSF dynamics are now recognized as a complex interplay of cardiac and respiratory forces, morphology, osmotic pressure, hydrostatic pressure, and local regulatory factors, rather than a unidirectional process [21, 31]. In the aqueduct, oscillatory CSF flow often exceeds net flow [15, 21, 32], suggesting that pulsatile pressure fluctuations may contribute to ventricular changes in hydrocephalus [33]. However, the causal relationship between abnormal CSF circulation and ventricular enlargement remains unresolved [2, 8, 9, 13, 34] with uncertainty regarding which process occurs first.

By analyzing the relationships among aqueductal resistance, Ratio‐Area, and Ratio‐SV in healthy young adults, this study offers new insights into the interplay between ventricular morphology and CSF dynamics. No significant correlation was found between Ratio‐SV and Ratio‐Area or between SV‐aq and Area‐vent (Figure S2), contrasting with observations in patients with communicating hydrocephalus [5], where a positive correlation between SV‐aq and Area‐vent has been reported.

In addition, we found a significant negative correlation between aqueductal resistance and SV‐aq in healthy adults. A descriptive observation in four NPH patients also suggested reduced aqueductal resistance. Taken together, these findings indicate that, within the normal physiological range, aqueductal resistance serves to stabilize the amplitude of CSF stroke volume (SV‐aq) driven by transmantle pressure (Figure 4c).

These findings highlight the need for future studies to investigate whether abnormally reduced aqueductal resistance may contribute to altered CSF circulation and its potential relationship with ventricular enlargement.

## 5 Limitations

First, the limited sample size may reduce statistical power, increasing the risk of Type II errors (false negatives) and result in instability, as individual data points may disproportionately influence outcomes. Findings with marginal significance (0.1 < *p* < 0.05), such as sex differences in Area‐vent and aqueductal resistance and sex‐specific correlations between aqueductal resistance and Ratio‐Area, should be interpreted cautiously. Future studies with larger cohorts are needed to improve statistical robustness and to confirm whether these patterns truly reflect sex‐related effects or are due to sample variability.

Second, the narrow age range of the study population limits the generalizability of these findings, which primarily serve as baseline reference data for young healthy individuals. Consequently, this study does not provide sufficient insights into age‐related changes in ventricular morphology or CSF dynamics, warranting further investigation in broader age groups. In addition, the NPH group was considerably older than the healthy group, and their data were included only for illustrative comparison, not for statistical testing.

Third, we acknowledge that the current findings are observational and based on a cross‐sectional analysis of healthy young adults. The role of aqueductal resistance in the early development of hydrocephalus remains unknown. To further investigate this mechanism, longitudinal studies or large‐scale cross‐sectional studies involving larger or more diverse populations—particularly elderly individuals or patients with evolving ventricular changes—will be required.

## 6 Conclusion

This study introduces two novel ratio‐based parameters, Ratio‐Area and Ratio‐SV, which reduce individual anatomical variations and provide stable metrics for assessing ventricular morphology and CSF dynamics. In healthy individuals, Ratio‐SV and Ratio‐Area are uncorrelated, while aqueductal resistance significantly correlates with Ratio‐SV.

These observations are consistent with the hypothesis that CSF circulation abnormalities related to reduced aqueductal resistance may play a role in the early stages of ventricular enlargement.

These results may provide a baseline reference for the clinical application of these novel metrics and offer new insights into the pathogenesis and potential therapeutic strategies for NPH.

## Supporting information


**Data S1:** jmri70139‐sup‐0001‐Supinfo.docx.
