# Supplementary material for: Relationships Between Lateral Ventricle Size, Cerebrospinal Fluid Dynamics, and Aqueductal Resistance in Young Healthy Adults
Source: J Magn Reson Imaging. 2025 Oct 3;63(3):721–32. doi: 10.1002/jmri.70139 (PMC12891764; doi:10.1002/jmri.70139)
Supplement: Supplementary file 1 — Data S1: jmri70139‐sup‐0001‐Supinfo.docx. [file JMRI-63-721-s001.docx]

Supplementary

## S1. Workflow of Area-vent binarization.

A comparison between Supplementary Figure S1 and Figure 4 allows the evaluation of whether ratio-based parameters and their corresponding raw values exhibit similar correlation patterns with aqueduct resistance. While some relationships were consistent across both analyses, others differed.


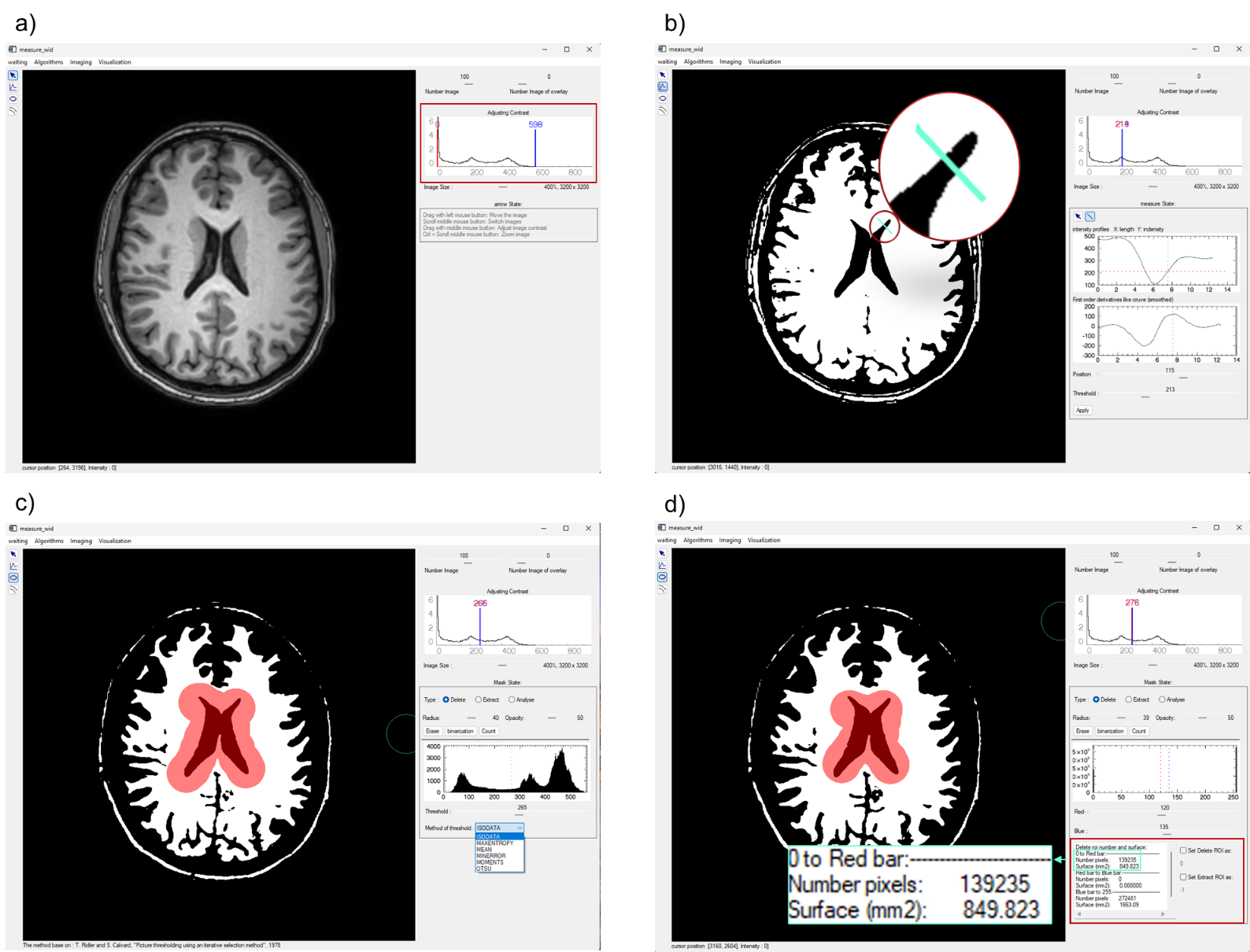


Figure S1 **Workflow of semi-automatic segmentation for ventricular area (Area-vent). a)** original interface view. The red box highlights the contrast adjustment bars: the red bar maps all lower-intensity pixels to the red threshold value, while the blue bar maps higher-intensity pixels to the blue threshold value. **b)** A line is drawn manually across the narrowest part of the ventricle; the software automatically selects the threshold based on the point of maximum intensity gradient along the line. **c)** One of six built-in automatic binarization algorithms is applied to define the threshold. **d)** Manual threshold selection by adjusting the contrast bars, which is the most commonly used method. The red-framed box displays the number of pixels and area within the ROI (shown in red). Note: The area of the choroid plexus is recorded separately and is not included in Area-vent.

## S2. Relationships between Area-vent, Area-brain, SV-aq, SV-cv and Resistance

A comparison between Supplementary Figure S1 and Figure 4 allows the evaluation of whether ratio-based parameters and their corresponding raw values exhibit similar correlation patterns with aqueductal resistance. While some relationships were consistent across both analyses, others differed.


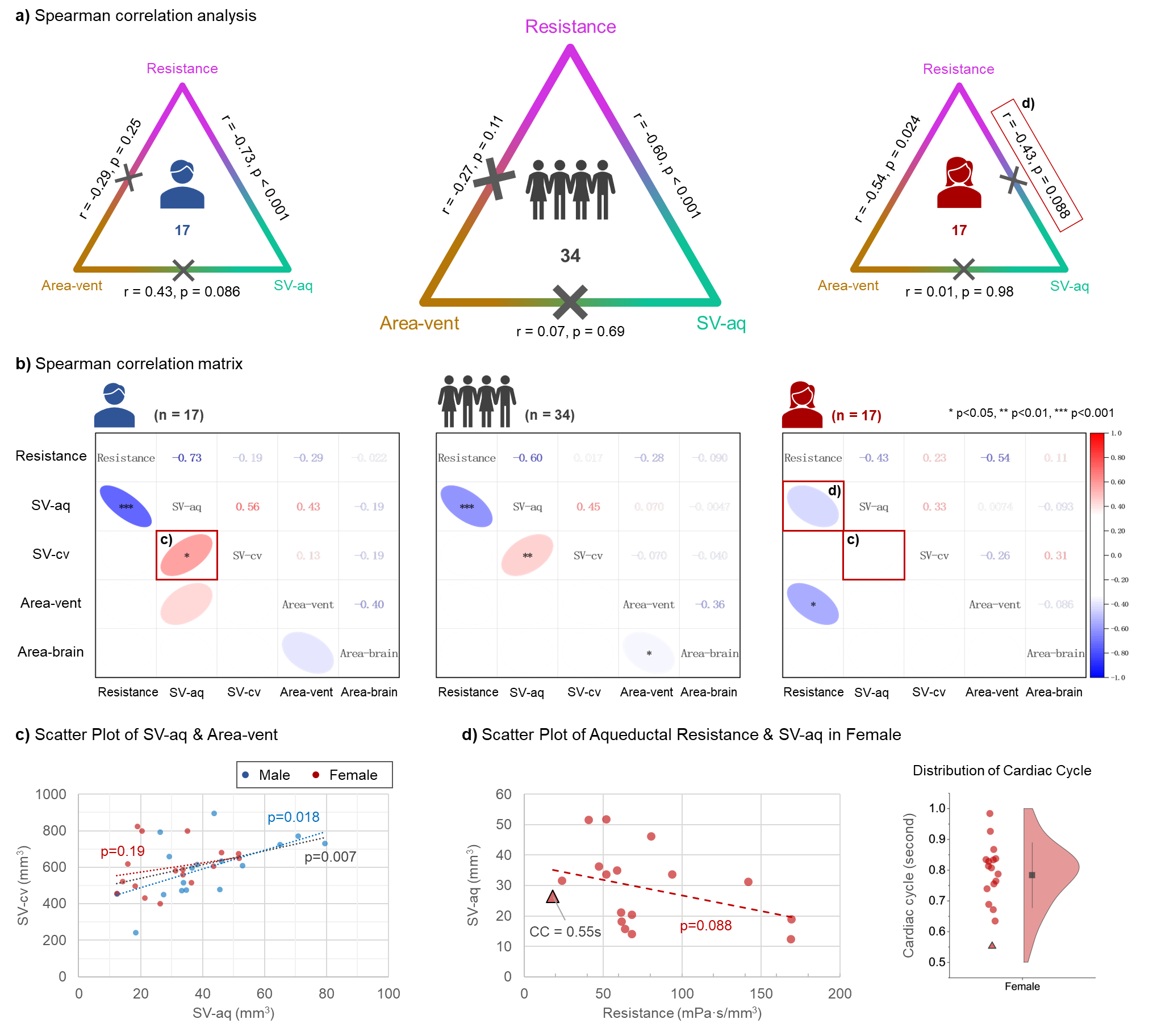


Figure S2 **Relationships among aqueductal resistance and original anatomical and flow parameters (Area-vent, Area-brain, SV-aq, and SV-cv) in male (left), combined (middle), and female (right) participants. a)** Triangular schematic showing the presence or absence of significant correlations among the three key parameters (Resistance, Area-vent, SV-aq), similar to Figure 4. **b)** Spearman correlation matrix for the five parameters, presented separately for men (n = 19), all participants (n = 36), and women (n = 17). The upper triangle shows Spearman's rank correlation coefficient (r), and the lower triangle displays trend ellipses with asterisks indicating three levels of significance (p < 0.05, p < 0.01, p < 0.001). **c)** A Scatter plot of Area-vent versus SV-aq, stratified by sex. **d)** In females, scatter plot of SV-aq versus aqueductal resistance (left), and cardiac cycle duration distribution (right). The triangle indicates the same participant in both panels. Area-vent: cross-sectional area of the lateral ventricles; Area-brain: cross-sectional area of the brain, SV-aq: aqueductal CSF stroke volume; SV-cv: CSF stroke volume at the C2–C3 spinal level.

In the combined population (Figure S1a, middle panel), the correlation pattern among aqueductal resistance, Area-vent, and SV-aq was similar to that observed among aqueductal resistance, Ratio-Area, and Ratio-SV in the main analysis (Figure 4a). A significant negative correlation was found between aqueductal resistance and SV-aq (p < 0.001, r = –0.60), while the correlations between resistance and Area-vent (p = 0.11, r = –0.27) and between Area-vent and SV-aq (p = 0.69, r = 0.07) were not statistically significant.

When stratified by sex, the correlation patterns based on raw parameters were not entirely consistent with those observed using ratio-based parameters in Figure 4a. In females, the correlation between aqueductal resistance and SV-aq did not reach statistical significance (p = 0.088, r = –0.43).

These differences may reflect the influence of confounding factors not accounted for in the raw parameters, particularly the effect of cardiac cycle duration on SV-aq. For example, in Figure S1d, one female participant (indicated by a triangle) exhibited the shortest cardiac cycle duration, which likely contributed to a reduced SV-aq value and affected the correlation. When cardiac cycle duration was accounted for by using Ratio-SV, the negative correlation with aqueductal resistance became statistically significant (Figure 5).
